# A new report of *Nocardiopsis valliformis* strain OT1 from alkaline Lonar crater of India and its use in synthesis of silver nanoparticles with special reference to evaluation of antibacterial activity and cytotoxicity

**DOI:** 10.1007/s00430-016-0462-1

**Published:** 2016-06-09

**Authors:** Dnyaneshwar Rathod, Patrycja Golinska, Magdalena Wypij, Hanna Dahm, Mahendra Rai

**Affiliations:** 1 Department of Microbiology, Nicolaus Copernicus University, Lwowska 1, 87 100 Toruń, Poland; 2 Nanobiotechnology Lab, Department of Biotechnology, SGB Amravati University, Amravati, Maharashtra 444602 India

**Keywords:** Alkaliphilic actinobacteria, Biosynthesis, Silver nanoparticles, Antibacterial activity, Cytotoxicity

## Abstract

The authors report the biological synthesis of silver nanoparticles (AgNPs) by alkaliphilic actinobacterium *Nocardiopsis valliformis* OT1 strain isolated for the first time from Lonar crater, India. The primary detection of silver NPs formation was made by colour change from colourless to dark brown and confirmed by UV–Vis spectrum of AgNPs at 423 nm, specific for AgNPs. Further, AgNPs were characterized by nanoparticle tracking analysis, Zeta sizer, Fourier transform infrared spectroscopy (FTIR) and transmission electron microscopy (TEM) analyses. FTIR analysis showed the presence of proteins as capping agent. TEM analysis revealed the formation of spherical and polydispersed AgNPs within the size range of 5–50 nm. The antimicrobial activity of silver nanoparticles against *Escherichia coli*, *Klebsiella pneumoniae*, *Pseudomonas aeruginosa*, *Staphylococcus aureus* and *Bacillus subtilis* was evaluated. The AgNPs showed the maximum antibacterial activity against *B. subtilis* (Gram positive) and the minimum against *E. coli* (Gram negative). The minimal inhibitory concentration values of AgNPs for the tested bacteria were found to be in the range of 30–80 µg/mL. The AgNPs demonstrated higher antibacterial activity against all the bacteria tested as compared with the commercially available antibiotics. The cytotoxicity of biosynthesized AgNPs against in vitro human cervical cancer cell line (HeLa) demonstrated a dose–response activity. The IC_50_ value was found to be 100 µg/mL of AgNPs against cancer HeLa cell line.

## Introduction

Nowadays, the multidrug resistance in microbes is becoming a global problem, which is the leading cause of human death [1–8]. Therefore, there is a need to search for efficient, safe and affordable antimicrobials to tackle the problem [7]. Since ancient times, silver and its compounds are being used as antimicrobial agents in diseases caused by bacteria, fungi and viruses [9–12]. The potential antibacterial activity of silver nanoparticles (AgNPs) against Gram-positive and Gram-negative bacteria including multidrug resistant strains was reported by many researchers [13–16]. The formation of biofilm by bacteria is a major threat to the health. Such bacteria are embedded in a complex polymer matrix and develop resistance to the antibiotics. The biofilms protects the bacterial cells as compared to the free living cells. There are various reports on role of AgNPs against microbial biofilm [13, 17, 18].

Similarly, Gandhiraj et al. [19] reported a significant anticancer activity of biosynthesized silver nanoparticles against breast cancer cell line MCF-7. The authors suggested that the synthesized AgNPs can be used to develop potential anticancer agent and active pharma molecule. Manivasagan et al. [20] also found cytotoxic effect of AgNPs against HeLa cancer cell line. Supraja and Arumugam [21] analysed cytotoxic effect of AgNPs and reported that cytotoxicity increased at higher concentrations. AgNPs demonstrated higher toxicity to microorganisms, while showed lower toxicity to mammalian cells as compared with other metals [22].

Biological synthesis of silver nanoparticles by different microorganisms such as bacteria [23–26], fungi [7, 27]; algae [28–30], plants [31–34], actinomycetes [15, 20, 35] and myxobacteria [36] has been attempted by many researchers. Among these organisms, the synthesis of nanoparticles by actinobacteria has been less known [37]. The method of biosynthesis is easy and eco-friendly. The actinobacteria are the producers of medicinally important bioactive compounds, mainly antibiotics, and those isolated from extreme and unexplored environments are predicted to be a rich source of novel antimicrobial agents [38].

Lonar Lake is located at Lonar in Buldhana district, Maharashtra, India, which was created by a meteor impact during the Pleistocene epoch. This lake, which lies in a basalt impact structure, is both saline and alkaline in nature [39, 40].

New species of *Nocardiopsis*, viz., *N. valliformis* HBUM 20028 (T), was isolated and described for the first time by Yang et al. [41]. We isolated a new strain of *Nocardiopsis valliformis* OT1 from Lonar crater, Maharashtra, India.

The present study was aimed to: (1) isolate and identify a new strain of *Nocardiopsis valliformis* OT1 from extreme habitat of alkaline Lonar crater; (2) use *N. valliformis* OT1 strain for biogenic synthesis of silver nanoparticles; and (3) assess antibacterial and cytotoxic activity.

## Materials and methods

### Isolation of *N. valliformis* OT1 strain

The actinobacterium *N. valliformis* OT1 strain was isolated from soil collected from the rim of Lonar Crater Lake located at Lonar in Buldhana district, Maharashtra, India, by the serial dilution method described by Golinska et al. [42] on the Starch Casein Agar (SCA, [43]) supplemented with 5 % NaCl, at pH 8.5.

The pH of collected Lonar Lake soil was found to be 10.4. The isolate was maintained on halophilic nutrient agar (5 g yeast extract, 10 g casitone, glucose 5 g and 60 g NaCl [44]) slants at room temperature and as suspensions of mycelial fragments and spores in 20 % glycerol (v/v) at −80 °C for further study. The *N. valliformis* OT1 strain grew in the presence of 5–15 % (w/v) sodium chloride and from pH 7.0 to 12.0 (all growth tests were carried out on halophilic nutrient agar).

### Molecular identification and phylogenetic analysis of *N. valliformis* OT1 strain

The actinobacterium *N. valliformis* OT1 strain was identified on the basis of 16S rRNA gene sequence. DNA was isolated from biomass harvested following growth of the isolate at 27 °C for 7 days in ISP2 broth, pH 5.5 [45]. DNA was extracted using DNA extraction kit (Sigma) according to manufacturer’s protocol, albeit with lysozyme at 45 mg/mL and incubation overnight at 37 °C. The PCR amplification of the 16S rRNA gene was performed in a 100-µL reaction mixture containing 8 µL template DNA (100 ng), 10 µL PCR buffer (Bioline), 2 µL of each PCR primer (p27F, p1525R, each 10 mM [46], 3.2 µL dNTP mix (12.5 mM; Bioline), 6 µL MgCl_2_ (50 mM; Bioline), 2 µL 5 U Taq DNA polymerase (Bioline) and 66.8 µL sterile MilliQ water. The PCR amplifications were made with an initial denaturation step at 95 °C for 1 min, followed by 30 cycles of denaturation at 95 °C for 1 min, annealing for 1 min at 55 °C and an extension at 72 °C for 1 min; following these procedures there was a final extension step of 72 °C for 5 min. The amplified product was purified using PCR purification kit (Qiagen). The sequencing reactions were carried out by sequencing service of Institute of Biochemistry and Biophysics Polish Academy of Sciences, Warsaw, Poland.

The search for the closest phylogenetic neighbours based on 16S rRNA gene similarity was performed using the EzTaxon server (http://eztaxon-e.ezbiocloud.net/, [47]). Phylogenetic analyses were carried out using MEGA 6 [48] and PHYML [49] software packages. Phylogenetic trees based on the aligned sequences were inferred using the neighbour-joining [50], the maximum-likelihood [51], maximum-parsimony [52] tree-making algorithms. The root position of the unrooted tree was estimated using the 16S rRNA gene sequence of *Streptomonospora nanhaiensis* 12A09^T^.

### Synthesis of silver nanoparticles (AgNPs) from *N. valliformis* OT1 strain

*N. valliformis* OT1 strain was grown in 250-mL Erlenmeyer flasks containing 100 mL halophilic nutrient broth (pH 8.5) and incubated in the orbital shaker (150 rpm) at 27 ± 1 °C for 8 days. The biomass was harvested by centrifugation at 6000*g* for 10 min and washed thrice with sterile distilled water to remove the attached medium components. Then, the biomass was resuspended in 100 mL sterilized distilled water and incubated at 27 ± 1 °C for 48 h. Thereafter, the biomass was filtered through Whatman filter paper no. 1 in order to obtain cell-free filtrate. Later, the filtrate was additionally harvested by centrifugation and treated with 1 mM silver nitrate solution, and kept at room temperature for 2–3 days. The supernatant (without silver nitrate) was used as control. The synthesized AgNPs were collected by centrifugation (12,000*g* for 30 min). After centrifugation, the supernatant was removed and the AgNPs were dried at 40 °C overnight. The mass of dried silver nanoparticles was estimated in milligrams. For further studies nanoparticles were dissolved in sterile distilled water/broth to obtain desired concentrations.

### Characterization of silver nanoparticles

#### Visual detection

After treatment of cell filtrate with silver nitrate, the preliminary detection of biosynthesized silver nanoparticles was carried out by visual observation of colour change from colourless to dark brown, which indicates the formation of Ag-nanoparticles.

#### UV-visible spectroscopy analysis

Absorption spectrum of the reaction mixture was measured between 200- and 800-nm wavelength range by UV–Vis spectrophotometer (NanoDrop ND-2000, Thermo Scientific, USA).

#### Zeta potential analysis

To understand the stability of biosynthesized silver nanoparticles, the zeta potential was measured. Nanoparticle samples (25 µL) were diluted 10 times and then sonicated for 15 min at 20 Hz. The mixture was filtered through 0.22-µm filter and used for zeta potential measurement by Malvern Zetasizer 90 (ZS 90, Malvern Instruments Ltd, UK).

#### Fourier Transform Infrared Spectroscopy (FTIR) analysis

To determine the biomolecules responsible for the reduction of silver ions and stabilization of AgNPs in the solution, the FTIR analysis was carried out. The powder of synthesized AgNPs was combined with dry KBr in the ratio of 1:100. AgNPs were characterized by FTIR spectroscopy (PerkinElmer FTIR-2000, USA) in the range 4000–400 cm^−1^ at a resolution of 4 cm^−1^.

#### Nanotracking analysis (NTA)

The nanotracking analysis was performed to measure the average size of the synthesized AgNPs. Five microlitres of the nanoparticle sample was diluted with 2 mL of nuclease-free water, then injected into the sample chamber and observed through camera coupled with the nanoparticle tracking analyser NanoSight LM20 (Malvern Instruments Ltd, UK).

#### Transmission Electron Microscopy (TEM) Analysis

The size and morphology of the AgNPs were analysed by FEI Tecnai F20 X-Twintool (Fei, USA) transmission electron microscopy operating at an acceleration voltage of 100 kV. The sample was prepared on a carbon-coated copper grids (400 µm mesh size) by dropping a small amount of solution of AgNPs. The sample was then allowed to dry at room temperature prior to measurements. The obtained data were assessed by Statistica Software (StatSoft, USA).

### Antibacterial activity of AgNPs

#### Activity of AgNPs individually and in combination with antibiotics against bacteria using disc diffusion method

The biosynthesized AgNPs from strain *N. valliformis* OT1 were screened against Gram-positive bacteria, namely *Staphylococcus aureus* (ATCC6338) and *Bacillus subtilis* (PCM2021 = ATCC 6633), and Gram-negative bacteria including *Escherichia coli* (ATCC8739), *Pseudomonas aeruginosa* (ATCC10145) and *Klebsiella pneumoniae* (ATCC700603) by disc diffusion method on Trypticase soy agar (TSA, Becton–Dickinson). The 100 µL of bacterial inoculum (1 × 10^6^ CFU/mL) was spread on to the surface of medium with sterile spreader. Subsequently, sterile disc (Ø 5 mm, Oxoid) impregnated with silver nanoparticles (20 µL) and standard antibiotic discs (kanamycin 30 mcg/disc, ampicillin 25 mcg/disc and tetracycline 30 mcg/disc, Oxoid) were placed on to the surface of medium inoculated with tested bacteria. Similarly, the combined effects of each standard antibiotic and AgNPs were determined. Prior to study, the antibiotic discs were impregnated with a concentrated colloidal solution of silver nanoparticles (20 µL). The cell-free filtrate was used as a control. The plates were incubated at 37 °C for 24 h, and zones of bacterial growth inhibition were measured (in mm). The assay was performed in triplicate.

#### Determination of MIC of antibiotics against tested bacteria

The minimum inhibitory concentration (MIC) is defined as the lowest concentration of chemicals that inhibits the growth of the organism. The MIC assays of antibiotics (ampicillin, kanamycin or tetracycline) were performed using Etest strips (BioMerieux) in the range of 0.016–256 µg/mL against bacterial isolates. The study was performed by diffusion method on Trypticase soy agar (TSA, Becton–Dickinson). The 100 µL of bacterial inoculum (1 × 10^6^ CFU/mL) was spread on to the surface of the medium with sterile spreader. Subsequently, Etest stripe was placed onto the surface of inoculated agar medium and incubated for 24 h at 37 °C. Assay was performed in triplicate.

#### Determination of the MIC of AgNPs against tested bacteria

The MIC was determined by using 96-well culture plates. The synthesized AgNPs were screened for MIC by microtitre broth dilution method in triplicate. Trypticase soy broth was used as diluents for bacterial strains. The final concentration of bacteria in each well was 1 × 10^6^ CFU/mL. The different concentrations (from 10 to 100 µg/mL) of AgNPs were used. The positive and negative controls were maintained. The microtitre plates were read at 450 nm on multimode reader (Biolog, USA) after incubation to determine the MIC values. Plates were incubated at 37 °C for 24 h.

#### Activity of AgNPs against bacteria in combination with antibiotics by dilution plate method

The MIC values of AgNPs from strain *N. valliformis* OT1 and commercial antibiotic were used to estimate accurate synergistic effect of AgNPs on antibiotic (kanamycin, ampicillin and tetracycline) activity. The assay was performed using 96-well culture plates in triplicate. The final concentration of bacteria in each well was 1 × 10^6^ CFU/mL. The Trypticase soy broth (TSB, Becton–Dickinson) was used as diluent for bacterial strains, antibiotics and AgNPs. The positive and negative controls were maintained. The microtitre plates were read at 450 nm on multimode reader (Biolog, USA) after incubation to determine the bacterial growth inhibition (%). Plates were incubated at 37 °C for 24 h.

### Cytotoxicity bioassay

Cell viability was evaluated by the MTT colorimetric technique using human HeLa cancer cell line, which was seeded in 96-well tissue culture plates. The monolayer cell culture was trypsinized, and the cell count was adjusted to 3 × 10^5^ cells/mL using medium containing 10 % newborn calf serum. To each well in microtitre plates, 100 µL of diluted cell suspension was added and incubated 24 h to form the cell monolayer. The supernatant was then flicked off, and 25 µL of AgNPs at final concentration 25, 50, 75 and 100 µg/mL was added to the cells in microtitre plate and incubated at 37 °C in 5 % CO_2_ incubator for 48 h. The cells were periodically checked for granularity, shrinkage and swelling. The sample solution in wells was then flicked off, and 25 μL of MTT (3-(4, 5-dimethylthiazolyl-2)-2,5-diphenyltetrazolium bromide) dye was added to each well for reduction of MTT by metabolically active cells. The plates were gently shaken and incubated for 4 h at 37 °C in CO_2_ incubator. The supernatant was removed and replaced with 100 µL of DMSO for solubilization of the MTT crystals. The absorbance was measured using a microplate reader (Biolog, USA) at a wavelength of 570 nm. First the percentage growth inhibition was calculated using following formula, % cell inhibition = 100 − {(At − Ab)/(AcAb)} × 100, where At = Absorbance value of test compound, Ab = Absorbance value of blank, Ac = Absorbance value of control. Then, % of cell viability = 100 %—% of cell inhibition was calculated. The IC_50_ value was plotted by taking the concentration of AgNPs on X-axis versus percentage of cell viability on Y-axis.

### Statistical analysis

To determine whether there are any significant differences among the activities of AgNPs, antibiotics and the combination of AgNPs and antibiotics, we applied ‘one-way ANOVA’ for the relative variability within above parameters.

## Results

### Molecular identification and phylogenetic analysis of *N. valliformis* OT1 strain

**Fig. 1:**
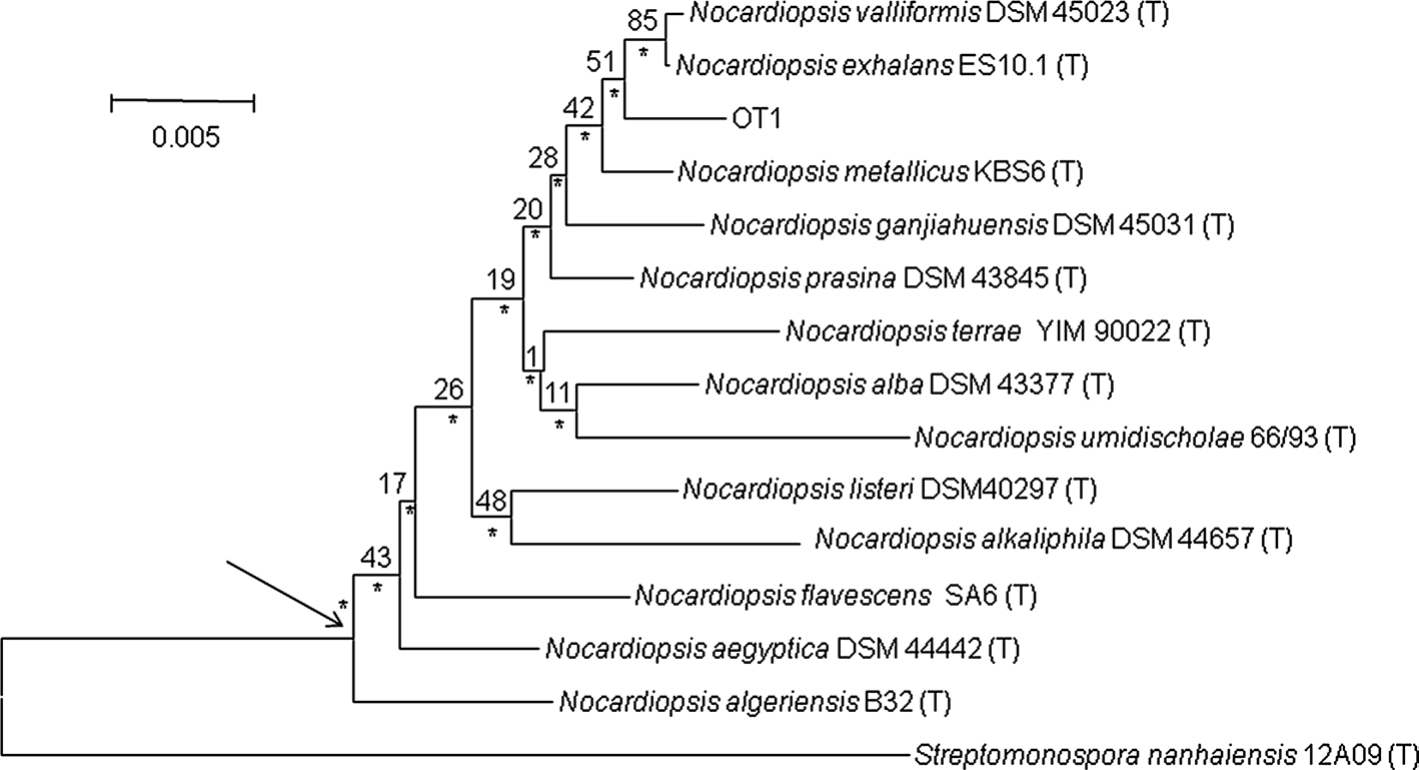
Neighbour-joining tree based on nearly complete 16S rRNA gene sequences (1430 nt) showing relationships between the isolate *N. valliformis* and the type strains *Nocardiopsis* species. Asterisks indicate branches that were also found using the maximum-likelihood and maximum-parsimony tree-making algorithms. Numbers at the nodes are percentage bootstrap values based on 1000 re-sampled datasets. T type strain. *Bar* 0.005 substitutions per nucleotide position. The root position of the tree was determined using *Streptomonospora nanhaiensis* 12A09^T^ as outgroup

Nearly complete 16S rRNA gene sequence of *N. valliformis* OT1 strain (1430 nt; GenBank accession number: KU523974) was determined. Based on the EzTaxon-e analysis, *N. valliformis* OT1 strain was affiliated to the genus *Nocardiopsis*, being most closely related to *N. valliformis* DSM-45023^T^ (99.4 %), *N. exhalans* ES10.1^T^ (99.4 %) and *N. metallicus* KBS6^T^ (99.4 %) and showed 8, 9 and 8 nt differences per 1430, 1428 and 1430 locations, respectively. 16S rRNA gene sequence similarities between *N. valliformis* strain OT1 and other type strains of the genus *Nocardiopsi*s were lower than 98.8 %. The phylogenetic trees based on 16S rRNA gene sequences showed that *N. valliformis* strain OT1 formed a distinct branch with *N. valliformis* DSM-45023^T^, *N. exhalans* ES10.1^T^ and *N. metallicus* KBS6^T^, which was supported by a bootstrap value of 42 % in the neighbour-joining tree (Fig. 1) and also recovered with the maximum-likelihood and maximum-parsimony algorithms.

### Characterization of AgNPs

**Fig. 2:**
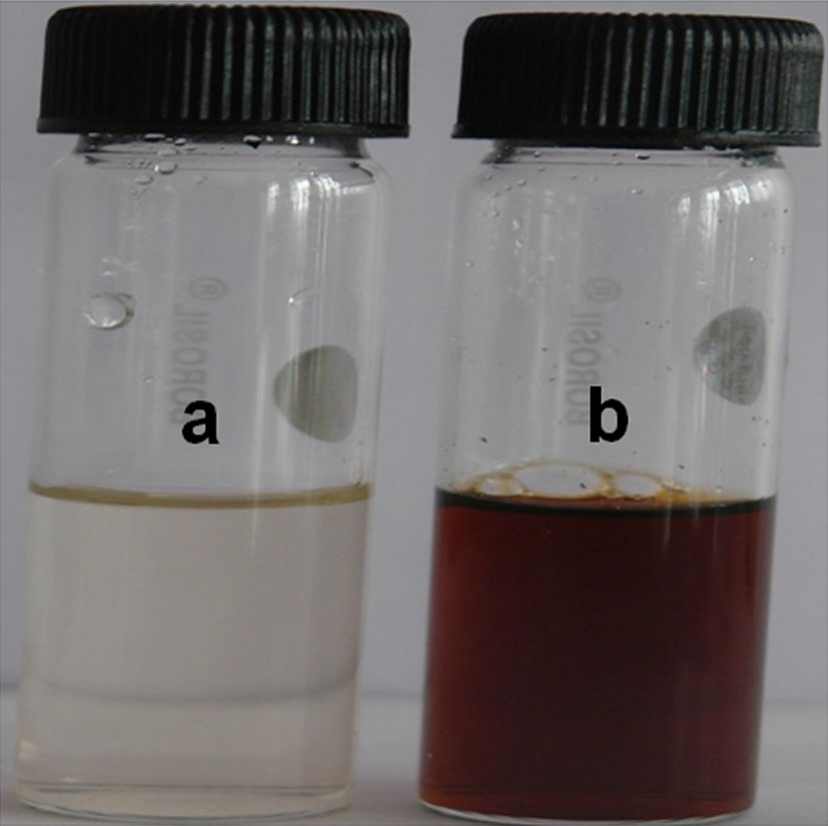
Visual detection of silver nanoparticles synthesized from *Nocardiopsis valliformis* OT1 strain control (**a**) and experimental (**b**)

**Fig. 3:**
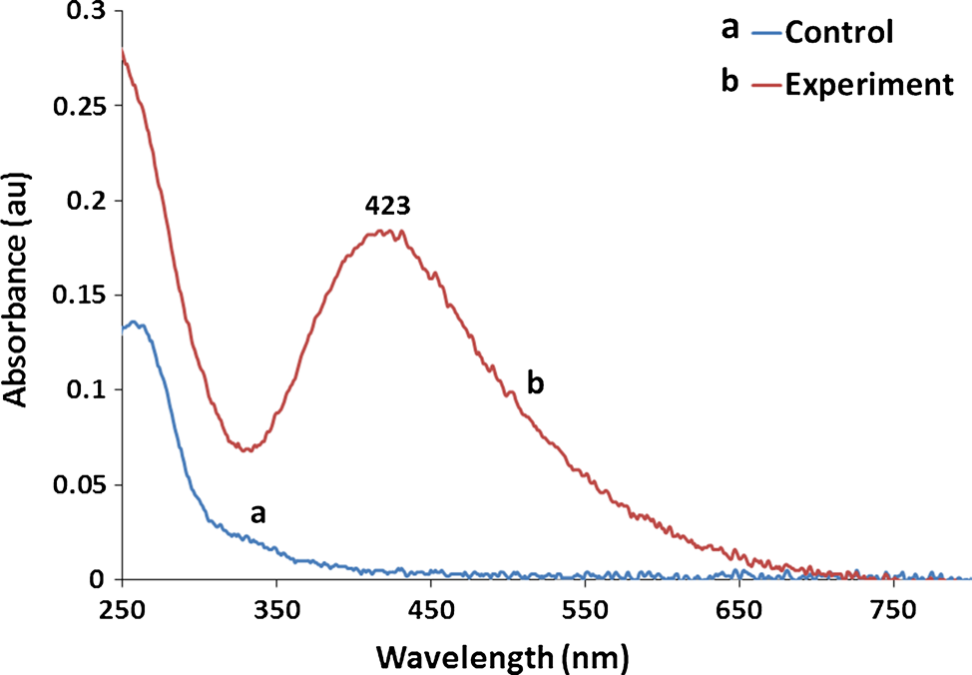
UV–Vis spectrum of silver nanoparticles synthesized from *Nocardiopsis valliformis* OT1 strain control (*a*) and experimental (*b*)

In the present study, silver nanoparticles (AgNPs) were synthesized by cell filtrate of actinobacterial *N. valliformis* OT1 strain which turned from colourless to dark brown after treatment with 1 mM AgNO_3_ (Fig. 2). Moreover, the biosynthesized Ag-nanoparticles were characterized by UV–Vis spectroscopy, which showed sharp narrow peak with a maximum absorbance at 423 nm (Fig. 3). The 25 mg of AgNPs was obtained from 100 mL of cell-free filtrate treated with 1 mM AgNO_3_.

**Fig. 4:**
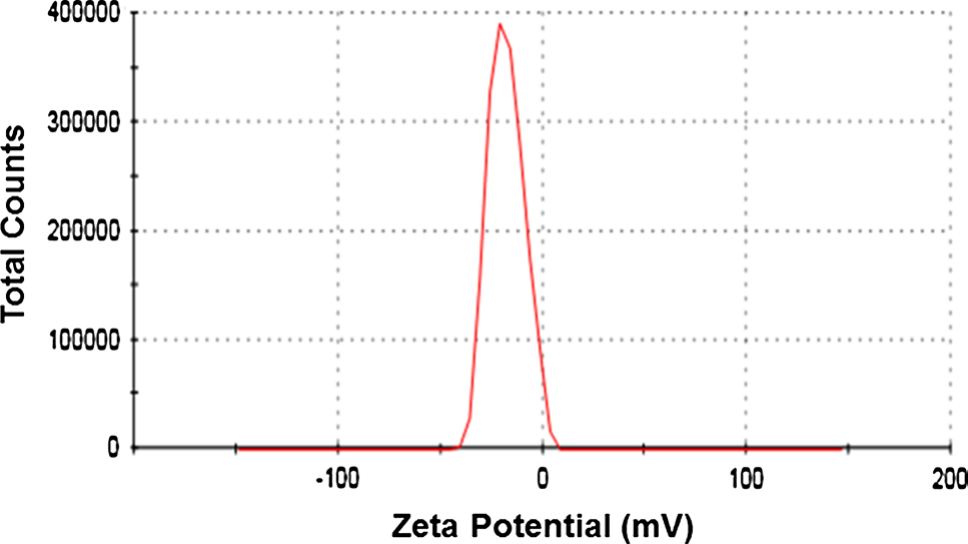
Zeta potential graph of silver nanoparticles synthesized from OT1 strain (−17.1 mV) at pH 7

The zeta potential of silver NPs synthesized from *N. valliformis* OT1 strain was found to be −17.1 mV which exhibited the stability of synthesized nanoparticles (Fig. 4).

**Fig. 5:**
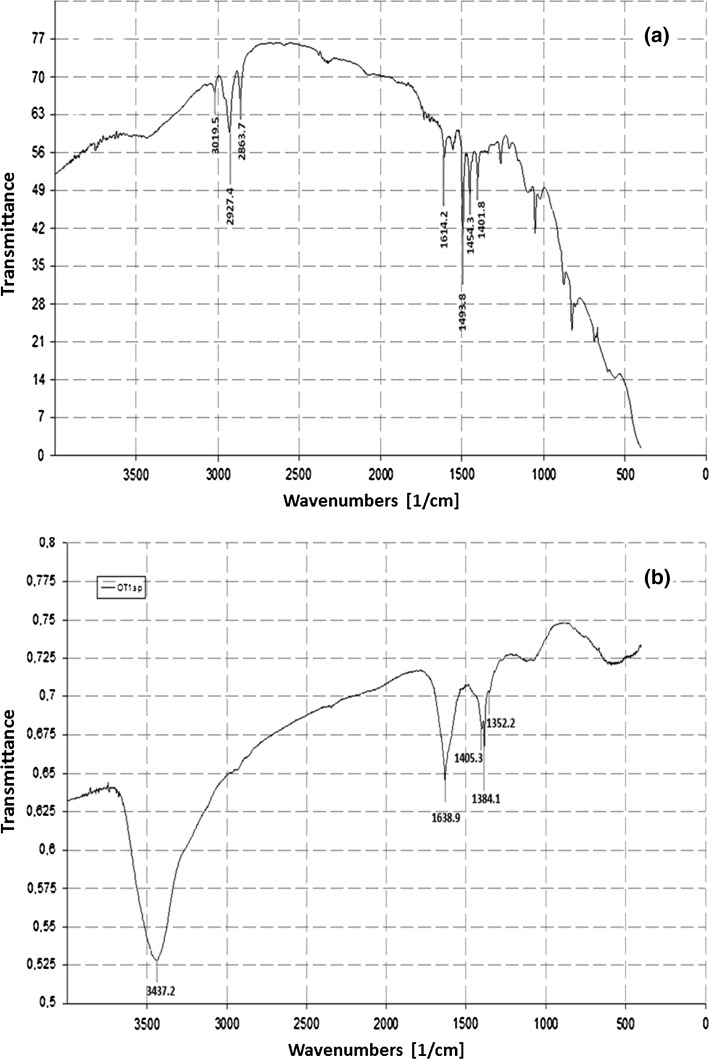
FTIR analysis of silver nanoparticles synthesized from OT1 strain. Control (**a**) and experimental (**b**)

The FTIR spectroscopy in the range 4000–400 cm^−1^ revealed the varied peaks, which corresponded to different functional groups and indicated the presence of stabilizing protein molecules on the surface of Ag-nanoparticles. In the present study, the Ag-nanoparticles synthesized from *N. valliformis* OT1 strain revealed absorbance peaks at 3437, 1639, 1405, 1384 and 1352 cm^−1^ (Fig. 5).

**Fig. 6:**
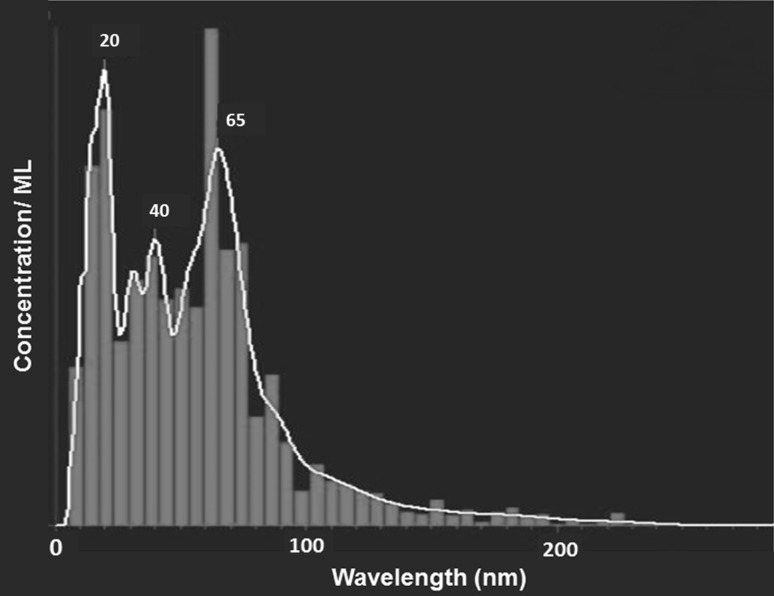
NTA of silver nanoparticles synthesized from *Nocardiopsis valliformis* OT1 strain

The NTAs showed mean size of Ag-nanoparticles synthesized from isolate *N. valliformis* OT1 of 62 (±51) nm (Fig. 6). The concentration of synthesized AgNPs was found to be 0.24 × 10^8^ particles/mL.

**Fig. 7:**
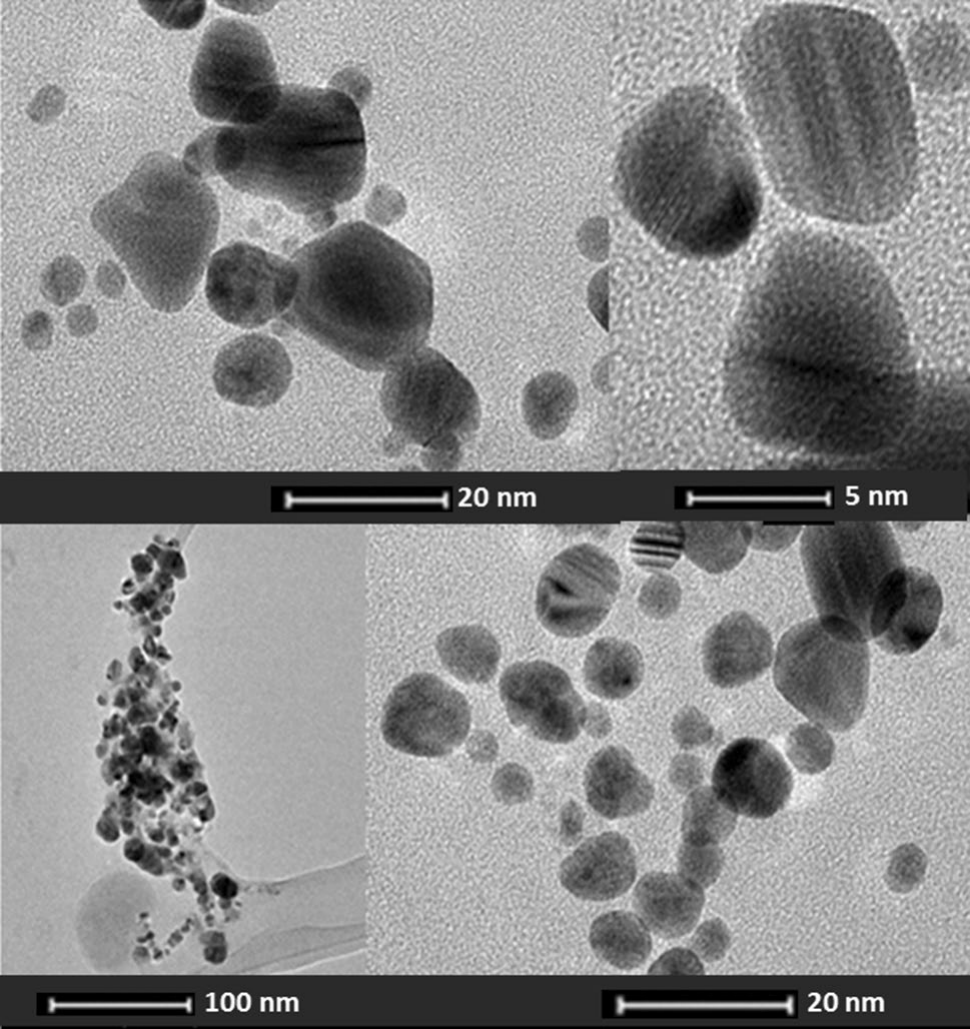
Transmission electron micrograph and selected area diffraction pattern of silver nanoparticles synthesized from *Nocardiopsis valliformis* OT1 strain

The TEM analysis of Ag-nanoparticles from *N. valliformis* OT1 strain showed the presence of spherical and polydispersed nanoparticles in the size range of 5–50 nm (Fig. 7). The biosynthesized Ag-nanoparticles were also found as aggregates at some places.

### In vitro activity of biogenic AgNPs against tested bacteria

The silver nanoparticles synthesized from *N. valliformis* were screened for its antibacterial activity against *Escherichia coli* (ATCC8739), *Staphylococcus aureus* (ATCC6338), *Klebsiella pneumoniae* (ATCC700603), *Pseudomonas aeruginosa* (ATCC10145) and *Bacillus subtilis* (PCM2021).

In the preliminary studies using disc diffusion method the Ag-nanoparticles showed maximum antibacterial activity against *B. subtilis* (13.66 mm), followed by *S. aureus* (12.16 mm), *P. aeruginosa* (11 mm), *K*. *pneumoniae* (10.16 mm) and *E. coli* (9.66 mm) (Table 1). The synergistic effect of silver NPs with antibiotics was also studied against above bacteria. The results of synergistic effects of the different antibiotics combined with synthesized AgNPs are shown in Table 1. Generally, the synergistic effect of AgNPs in combination with antibiotics was found to be with all tested antibiotics (with exception of AgNPs and ampicillin against *Pseudomonas aeruginosa*). The most interesting observations were noticed against *K*. *pneumoniae* and *P. aeruginosa*, which were resistant to antibiotics alone (ampicillin and kanamycin, respectively), but sensitive when antibiotics were combined with synthesized Ag-nanoparticles (Table 1).

**Table 1:** Antibacterial activity of AgNPs synthesized from *Nocardiopsis valliformis* strain OT1 and its synergistic effect with antibiotics (ø of disc 5 mm)

Bacteria	AgNPs	Kanamycin		Ampicillin		Tetracycline	
		A	A + AgNPs	A	A + AgNPs	A	A + AgNPs
*Escherichia coli* ATCC8739	9.66 ± 0.57	14.66 ± 1.15	15.33 ± 0.57	16 ± 0.57	21.66 ± 0.57	19.33 ± 0.76	22.33 ± 0.57
*Klebsiella pneumoniae* ATCC700603	10.16 ± 1.04	NI	10.33 ± 0.57	NI	6 ± 0	11.83 ± 0.28	13.83 ± 0.28
*Pseudomonas aeruginosa* ATCC10145	11 ± 1	NI	10 ± 0	11.83 ± 0.28	9.83 ± 0.28	11.16 ± 0.76	16.16 ± 1.04
*Staphylococcus aureus* ATCC6338	12.16 ± 1.25	15.83 ± 0.28	16.83 ± 0.28	33.33 ± 1.52	40.5 ± 0.5	26.5 ± 0.5	28.83 ± 1.04
*Bacillus subtilis* PCM2021	13.66 ± 0.57	17.22 ± 0.26	19.5 ± 0.5	16.33 ± 1.04	18.25 ± .05	30.33 ± 0.57	32.5 ± 0.5

Inhibition zones in diameter (mm)

Mean value is significantly different at *p* ≤ 0.05

Values expressed in mean ± SD

A, antibiotic; AgNPs, silver nanoparticles; NI, no inhibition

The minimum inhibitory concentration (MIC) of biosynthesized Ag-nanoparticles was screened against all the tested bacteria. The MIC values of silver NPs were found to be in the range of 30–80 µg/mL (Table 2).

**Table 2:** MIC of antibiotics and AgNPs synthesized from *Nocardiopsis valliformis* strain OT1 and synergistic effect of AgNPs combined with antibiotics against bacterial isolates by dilution plate method

Bacteria	MIC of AgNPs (µg/ml)	MIC of antibiotics (µg/ml)			AgNPs^b^	Kanamycin^b^		Ampicillin^b^		Tetracycline^b^	
		Kan	Amp	Tet		A	A + AgNPs	A	A + AgNPs	A	A + AgNPs
*Escherichia coli* ATCC8739	80 ± 0.17	1.5 ± 0.5	2.0 ± 0.0	0.79 ± 0.35	61.1 ± 0.05	39.62 ± 0.01	62.16 ± 0.04	73.94 ± 0.06	72.61 ± 0.01	23.13 ± 0.02	61.1 ± 0.05
*Klebsiella pneumoniae* ATCC700603	70 ± 0.07	32.0 ± 4.6	Not found^a^	24.0 ± 4.61	36.81 ± 0.01	28.01 ± 0.23	72.61 ± 0.01	35.09 ± 0.03	78.73 ± 0.05	84.52 ± 0.15	84.83 ± 0.01
*Pseudomonas aeruginosa* ATCC10145	60 ± 0.04	32.0 ± 0.0	Not found^a^	21.3 ± 4.6	76.74 ± 0.01	33.21 ± 0.04	84.83 ± 0.01	9.91 ± 0.06	76.56 ± 0.01	81.66 ± 0.03	86.13 ± 0.01
*Staphylococcus aureus* ATCC6338	30 ± 0.11	1.16 ± 0.28	0.1 ± 0.03	0.25 ± 0.0	73.82 ± 0.02	39.54 ± 0.01	75.17 ± 0.09	86.68 ± 0.05	86.13 ± 0.05	44.2 ± 0.03	76.5 ± 0.05
*Bacillus subtilis* PCM2021	60 ± 0.14	29.3 ± 0.17	0.1 ± 0.01	0.15 ± 0.08	35.85 ± 0.05	85.9 ± 0.01	86.13 ± 0.01	34.62 ± 0.22	37.64 ± 0.03	17.14 ± 0.04	35.85 ± 0.05

Bacterial growth inhibition (%)

Mean value is significantly different at *p* ≤ 0.05

Values expressed in mean ± SD

MIC, minimal inhibitory concentration; Kan, kanamycin; Amp, ampicillin; Tet, tetracycline; A, antibiotic; AgNPs, silver nanoparticles

^a^For synergistic studies the highest concentration of antibiotic of Etest stripes was used (256 µg/mL)

^b^Growth inhibition (%) in the presence of MICs of AgNPs or/and antibiotic

The results of synergistic effect of AgNPs with antibiotics using plate dilution method are presented in Table 2. The maximum synergistic effect was observed for ampicillin and kanamycin when combined with AgNPs against *Pseudomonas aeruginosa* (41.92 and 35.8 %, respectively, higher bacterial growth inhibition than AgNPs used individually). Significant enhancement of bacterial growth inhibition was observed in the presence of AgNPs and kanamycin against *Pseudomonas aeruginosa* (>8 %).

### Cytotoxicity bioassay

**Fig. 8:**
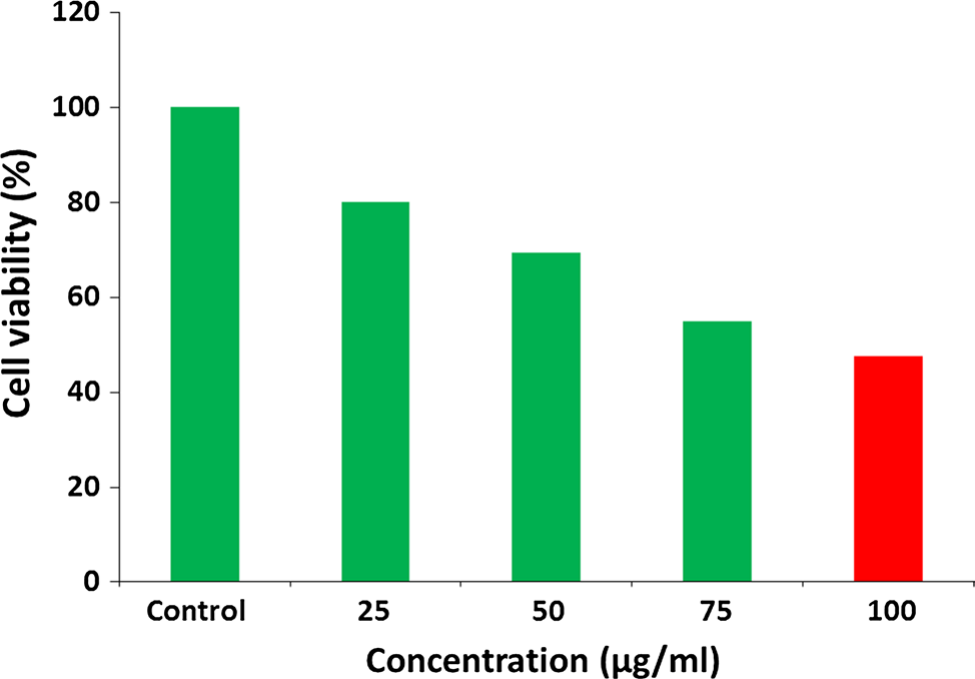
Cytotoxicity of synthesized AgNPs from *N. valliformis* OT1 actinobacterial strain against cancer HeLa cell line

The in vitro cytotoxic activity of bioreduced AgNPs from *N. valliformis* OT1 strain was screened against cancer HeLa cell line. Encouragingly, AgNPs having concentrations of 25, 50, 75 and 100 µg/mL showed 19.87, 30.72, 45.18 and 52.40 % inhibition against cancer HeLa cell line, respectively (Fig. 8).

## Discussion

Since Yang et al. [41] have reported new species of *N. valliformis* from alkaline lake of China, it is a first report on isolation of alkaliphilic actinobacterium *Nocardiopsis valliformis* OT1 strain from Lonar crater of Central India and its application in synthesis of AgNPs and antibacterial activity against bacterial pathogens [41].

The synthesis of AgNPs was studied after the treatment of cell-free filtrate with silver nitrate solution. The reduction of Ag ions into AgNPs is indicated by the change in colour from colourless to dark brown and the absorbance property of AgNPs at about 420 nm of wavelength. The presence of specific peak is due to the surface plasmon resonance property of noble metal nanoparticles [14, 16, 53].

The zeta potential is a measure of the electrostatic potential on the surface of the nanoparticles and is related to the electrophoretic mobility and stability of colloidal suspension of nanoparticles [54]. It has been reported that the particles with higher negative or positive zeta potential value possess a force to repel each other and do not form aggregates. Unlike these with low zeta potential value, which has no force to avoid particles coming together, particles form a bigger particle as aggregation [55]. Our findings are similar to results by Gaikwad et al. [56] and Rai et al. [55] who reported zeta potential value range from −5.31 to −15.8 mV of mycogenic AgNPs.

To understand the contribution of biomolecules responsible for the reduction of the Ag^+^ ions into Ag-nanoparticles and the presence of capping agents over the bioreduced AgNPs responsible for stabilization of AgNPs, the FTIR spectroscopy was performed. The absorbance band at 3437 cm^−1^ can attribute to the vibrations of amino groups (N–H) related to the presence of peptides [7, 16, 57]. However, the peak at 1639 cm^−1^ may be because of carboxylic group (C=O) stretching. Similarly, the absorbance at 1405 cm^−1^ can be assigned to C–H asym. deformation vibration [20]. In addition, the peak at 1384 cm^−1^ is associated with CH_3_ sym. bending and at 1352 cm^−1^ is resemblance to the C–H deformation vibration [58]. From the above results, it is assumed that capping agent as proteins binds to silver NPs. Gole et al. [59] reported that proteins are responsible for binding to silver nanoparticles by the electrostatic attraction of negatively charged carboxylate groups present in the protein secreted by fungus. Consequently, the AgNPs become stable by proteins [59].

The average size of biosynthesized Ag-nanoparticles was measured by nanoparticle tracking analysis (NTA). NTA of AgNPs is based on light scattering and Brownian motion properties in order to obtain the good sizing accuracy and relatively narrow distributions from monodispersed samples [60]. The similar size of silver nanoparticles (68.1 nm) was found by Chauhan et al. [61] who studied Ag-nanoparticles synthesized from *Streptomyces* sp.

The TEM analysis showed that the actinobacterial strain *N. valliformis* OT1 was capable of synthesizing small-sized AgNPs. The present observations were found to be similar to other previous reports [20, 62, 63] who found spherical AgNPs with some aggregations. The synthesis of small-sized (5–40 nm) and spherical silver nanoparticles was also reported by Sukanya et al. [64] who studied actinomycete culture of *Streptomyces* sp. II isolated from heavy metal-polluted and non-polluted areas in India. The difference in size of AgNPs analysed by TEM and NTA is due to the fact that TEM analyses reveal exact size and image of the metal nanoparticles as the beam of electrons used. It transmits electrons through an ultrathin specimen, which interacts with the sample, whereas in the NTA hydrodynamic radius is calculated, which is always larger. Therefore, the size measurement results using NTA are bigger when compared to TEM.

The mechanisms of antibacterial effect of silver nanoparticles are still unclear. The most widely known mechanism of AgNPs is the inhibition of the enzymatic function of some proteins by binding to the thiol groups of l-cysteine [65]. It is claimed that AgNPs promote the permeability of the bacterial membrane and disrupt the membrane integrity [20, 22, 66] which are also thought to be responsible for the antibacterial effect. Some studies showed that silver can bind to DNA, which increases the decomposability of genome DNA [67]. Silver NPs may also inactivate the respiratory chain and be cause of hydroxyl radicals’ formation [65].

Depending on the size of AgNPs, its large surface area comes in contact with bacterial cell [2, 7, 68]. The smaller the size of silver nanoparticles, the larger the surface area to volume ratio, and hence obviously the bactericidal activity of silver nanoparticles is affected by the size of nanoparticles. Panacek et al. [68] studied the bactericidal activity based on the size of nanoparticles (27, 37, 46 and 52 nm) and observed that nanoparticles having 25 nm showed highest antibacterial activity. The antibacterial activity of silver nanoparticles also depends upon its shape [69].

Synergistic effect of antibiotics and silver nanoparticles to Gram-positive and Gram-negative bacteria provided helpful insight into the development of new antimicrobial agents with the enhancement of the antibacterial mechanism against pathogenic microorganisms. Such a mechanism was observed against almost all the tested bacterial pathogens after using disc diffusion method. However, the highest synergistic effect was observed for *K*. *pneumoniae* and *P. aeruginosa* when biosynthesized AgNPs from *N. valliformis* OT1 strain were combined with antibiotics. The bacteria were resistant to antibiotics alone (ampicillin and kanamycin, respectively). However, the application of biosynthesized AgNPs together with antibiotic enhanced the activity of ampicillin and kanamycin leading to inhibition of bacterial growth.

After using the dilution plate method the enhancement of growth inhibition of tested bacteria was variable when combination of AgNPs and antibiotics was used. The significant synergistic effect of AgNPs in combination with ampicillin or kanamycin was noticed against *Klebsiella pneumoniae* (41.92 and 35.8 %, respectively). The 8.09 % enhancement of growth inhibition was observed in the presence of AgNPs and ampicillin against *Pseudomonas aeruginosa.* These findings support results of preliminary studies which have been carried out by disc diffusion method. Shahverdi et al. [70] who studied the combined effect of AgNPs and antibiotics found increase in antibacterial activity of antibiotics against bacterial cells. They claimed that synergism was caused by binding reaction between antibiotic molecules which showed hydroxyl and amino groups that may easily react with AgNPs. Similarly, our findings support results of Li et al. [71] who reported the synergistic antibacterial effects of antibiotic (amoxicillin) and silver nanoparticles.

The results of minimal inhibitory concentration confirmed that silver nanoparticles biosynthesized from *N. valliformis* OT1 strain were active against tested bacteria. Singh et al. [72] when studied MIC of AgNPs synthesised from *Acinetobacter calcoaceticus* reported activity of AgNPs against human pathogenic bacteria in much higher concentration range of 150–600 μg/mL as compared to our findings.

Cell culture-based assays are used as a pre-screening tool to understand the biological effects of nanoparticles. To detect the viability of cells, the method of Mosmann using the MTT colorimetric assay was performed. This assay is for measuring the activity of enzymes that reduce MTT or close dyes (XTT, MTS) to formazan, giving purple colour. The main application allows assessing the viability (cell counting) and the proliferation of cells (cell culture assay). It can be used to determine cytotoxicity of potential agent and toxic material, since those agents would stimulate or inhibit cell viability and growth [73].

The in vitro cytotoxic activity of silver nanoparticles was determined by MTT assay against cancer HeLa cells. We observed that the synthesized AgNPs inhibited the cell growth of cancer HeLa cell line. The biosynthesized AgNPs induced cytotoxicity in HeLa cell line in lower concentration (IC_50_ at 100 µg/mL of AgNPs) when compared to results by Manivasagan et al. [20] who reported the IC_50_ of HeLa cell line at concentration of 200 µg/mL of biosynthesized AgNPs from *Nocardiopsis* sp. MBRC-1.

*N. valliformis* strain OT1 was found to be simple, easy and eco-friendly system for the biological synthesis of AgNPs. The FTIR analysis showed the presence of protein as capping agent, which is responsible for stability of nanoparticles. The AgNPs were found to be small in size which may influence on its higher antibacterial activity [74]. Encouragingly, biosynthesized Ag-nanoparticles demonstrated remarkable antibacterial activity against bacterial pathogens as compared to commercially available antibiotics. On the basis of obtained results it seems that the combination of antibiotics with AgNPs against Gram-positive and Gram-negative bacteria offers a valuable contribution to nanomedicine. Similarly, the biogenic AgNPs demonstrated considerable cytotoxic effect on cancer HeLa cell line. Moreover, it can be suggested that the extreme habitats such as alkaline Lonar crater are still unexplored source of microorganisms which may produce bioactive compounds.
